# Diagnosis of multiple sclerosis using multifocal ERG data feature fusion

**DOI:** 10.1016/j.inffus.2021.05.006

**Published:** 2021-12

**Authors:** A. López-Dorado, J. Pérez, M.J. Rodrigo, J.M. Miguel-Jiménez, M. Ortiz, L. de Santiago, E. López-Guillén, R. Blanco, C. Cavalliere, E. Mª Sánchez Morla, L. Boquete, E. Garcia-Martin

**Affiliations:** a Biomedical Engineering Group, Department of Electronics, University of Alcalá, Alcalá de Henares, Spain; b Department of Ophthalmology, Miguel Servet University Hospital, Zaragoza, Spain; c Aragon Institute for Health Research (IIS Aragon). Miguel Servet Ophthalmology Innovation and Research Group (GIMSO), University of Zaragoza, Spain; d School of Physics, University of Melbourne, VIC 3010, Australia; e Department of Surgery, Medical and Social Sciences, University of Alcalá, Alcalá de Henares, Spain; f RETICS: Thematic Networks for Co-operative Research in Health for Ocular Diseases, Spain; g Department of Psychiatry, Hospital 12 de Octubre Research Institute (i+12), 28041 Madrid, Spain; h Faculty of Medicine, Complutense University of Madrid, 28040 Madrid, Spain; i CIBERSAM: Biomedical Research Networking Centre in Mental Health, 28029 Madrid, Spain

**Keywords:** Multiple sclerosis, Multifocal electroretinogram, Feature fusion, Continuous wavelet transform, Empirical Mode Decomposition, Support vector machine

## Abstract

The purpose of this paper is to implement a computer-aided diagnosis (CAD) system for multiple sclerosis (MS) based on analysing the outer retina as assessed by multifocal electroretinograms (mfERGs). MfERG recordings taken with the RETI‐port/scan 21 (Roland Consult) device from 15 eyes of patients diagnosed with incipient relapsing-remitting MS and without prior optic neuritis, and from 6 eyes of control subjects, are selected. The mfERG recordings are grouped (whole macular visual field, five rings, and four quadrants). For each group, the correlation with a normative database of adaptively filtered signals, based on empirical model decomposition (EMD) and three features from the continuous wavelet transform (CWT) domain, are obtained. Of the initial 40 features, the 4 most relevant are selected in two stages: a) using a filter method and b) using a wrapper-feature selection method. The Support Vector Machine (SVM) is used as a classifier. With the optimal CAD configuration, a Matthews correlation coefficient value of 0.89 (accuracy = 0.95, specificity = 1.0 and sensitivity = 0.93) is obtained. This study identified an outer retina dysfunction in patients with recent MS by analysing the outer retina responses in the mfERG and employing an SVM as a classifier. In conclusion, a promising new electrophysiological-biomarker method based on feature fusion for MS diagnosis was identified.

## Highlights

• A computer-aided system for diagnosis of multiple sclerosis is implemented.
• 40 features are obtained from the multifocal electroretinogram recordings.
• The four most relevant features are selected using a filter and the wrapper selection method.
• The classifier produces a Matthews correlation coefficient value of 0.89.
• A promising new electrophysiological-biomarker method for diagnosis of multiple sclerosis is identified.

## 1 Introduction

Multiple sclerosis (MS) is a degenerative disease that affects the central nervous system (CNS). It is characterized by the presence of inflammatory lesions and demyelination [1]. MS symptoms vary widely among patients: muscular, cognitive, visual, etc. The cause of MS is not known and there is no single biomarker with which to diagnose it or measure its development [2,3].

In 2013, the number of people worldwide diagnosed with MS stood at 2,500,000 [4]. In many countries, it is the second most prevalent cause of disability in adults, after traffic accidents. The male:female prevalence ratio is 1:2 and it often occurs in young individuals (20–40 years old).

Early treatment of MS has been associated with better clinical outcomes [5]. However, the chance of misdiagnosis is high when symptoms are vague and/or do not logically localize to the CNS, when neurological examination produces normal findings and when MRI abnormalities are nonspecific [6]. Consequently, successfully finding biomarkers is critical to enhancing accurate diagnosis of MS [7].

The current criteria (McDonald criteria) used to diagnose forms of MS were originally formulated by [8] and revised in [9], [10], [11]. Diagnosis should take into account evidence of damage to the CNS disseminated in time (on different dates) and in space (damage to at least two different parts of the CNS) and should exclude other conditions that, due to their clinical or laboratory profile, can mimic MS.

It is important to investigate new biomarkers that allow early diagnosis of MS and which provide information about both the progression of the disease and the efficiency of patient treatment. In the latest review of the McDonald criteria [11], the need to investigate visual-evoked potentials is highlighted. There is evidence that in a high percentage of cases (30–60% according to different authors), the first symptoms of MS manifest in the visual pathway. Evoked potentials are more closely related to clinical disability than structural data [12].

Visual electrophysiology may prove to be a critical tool in assessing response to MS therapies in clinical practice [13]. Increasingly, diseases affecting the brain are found to be associated with functional changes in the outer and inner retina neurons [14]. The electroretinogram (ERG) measures the electrical activity of photoreceptors (outer retina) from the entire retina in response to a light stimulus. In addition, some studies have shown the utility of the ERG in detecting MS [15,16].

The multifocal electroretinogram (mfERG) technique allows the individualized stimulation of a large number (typically 63–103) of sectors of the central retina (macula) using a pseudo-random stimulus (m-sequence) [17]. Depending on the visual stimulation paradigm, different types of response are evoked. The typical mfERG response (first-order kernel) is a biphasic wave consisting of a negative wave (N1) followed by a large positive response (P1). There may be other subsequent waves and even some oscillatory potentials. Outer retinal electroretinographic changes in MS patients have been reported previously [18], [19], [20], [21], [22], [23]. In all these papers, amplitude and latency were analysed using traditional methods and the findings varied substantially.

It is also possible to include other parameters that contribute to a better definition of the mfERG signal, such as slopes, amplitude differences between peaks, etc. [24]. Time–frequency analysis of mfERG responses using the wavelet transform has been tested for diagnosis of glaucoma, employing three versions of the former: continuous wavelet transform (CWT) [25], discrete wavelet transform (DWT) [26] [27] and wavelet packets [28].

It has recently been demonstrated that analysing mfERG recordings using an adaptive filter based on empirical mode decomposition (EMD), combined with use, as a feature, of the value of the correlation of the filtered signals with a normative database, obtains good discriminant values (evaluated in terms of area under the curve, AUC) between control subjects and MS patients [29].

Clinical decisions are often based on analysis of various information sources (medical imaging, biochemistry, genetics, etc.) and practitioners make their diagnosis from the fusion of this information, with computer-aided diagnosis (CAD) systems increasingly supporting clinical data and the presence of signs and symptoms. Use of CAD systems to support decision-making is becoming more and more common due to their widening reach, which now extends to most medical areas, and the high levels of accuracy achieved [30].

**Fig. 1:**
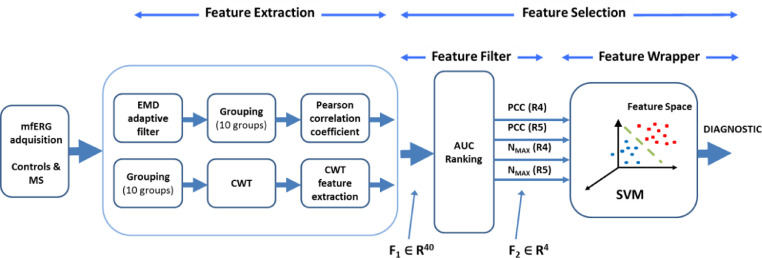
General block diagram of the CAD system. MS: Multiple sclerosis; mfERG: multifocal electroretinogram; EMD: empirical mode decomposition; CWT: continuous wavelet transform; AUC: area under the curve; SVM: support vector machine; PCC: Pearson correlation coefficient; N_MAX_: number of local maxima in the continuous wavelet domain; R4: Ring 4; R5: Ring 5, F_1_, F_2_: feature vectors.

MS: Multiple sclerosis; mfERG: multifocal electroretinogram; EMD: empirical mode decomposition; CWT: continuous wavelet transform; AUC: area under the curve; SVM: support vector machine; PCC: Pearson correlation coefficient; N_MAX_: number of local maxima in the continuous wavelet domain; R4: Ring 4; R5: Ring 5, F_1_, F_2_: feature vectors.

The aim of this study is to investigate automatic diagnosis of MS using a variety of fusioning features (Pearson correlation coefficient between the adaptively filtered signals using EMD and a normative database, CWT domain) from a single form of outer-retinal evoked potential (mfERG). A classifier (support vector machine, SVM) is used to perform automatic diagnosis, as per the block diagram shown in Fig. 1.

## 2 Methods

### 2.1 Subject database

The study procedures were performed in accordance with the tenets of the Declaration of Helsinki, and ethical approval was obtained from the local ethics committees [Clinical Research Ethic Committee of Aragon (CEICA, Zaragoza, Spain)]. All subjects were over the age of 18 and signed informed consent prior to study procedures.

This paper analyses the mfERG recordings of 15 patients and 6 control subjects. Patients were diagnosed by a specialist neurologist following the modified McDonald criteria [10] and they were all classified as relapsing-remitting MS (RRMS) during daily clinical care. Subjects who accepted the invitation to participate in the study were given a complete neuro-ophthalmological examination, which included assessment of best-corrected visual acuity using the Snellen chart, pupillary reflexes, ocular motility, examinations of the anterior segment, intraocular pressure (IOP) with the Goldmann applanation tonometer, and papillary morphology by funduscopic exam. This was performed on all subjects in order to detect ocular impairments (such as primary open-angle glaucoma, cataracts and corneal pathology) that might affect functional vision or mfERG results.

Participants were excluded from the study if they presented visual acuity < 0.6 (Snellen scale) or intraocular pressure > 20 mmHg. Participants had no concomitant ocular diseases, nor any previous history of retinal pathology, glaucoma or significant refractive errors (more than 5 dioptres of spherical equivalent refraction or 3 dioptres of astigmatism) nor systemic conditions that could affect the visual system. The results were obtained from the random selection of one eye from each of the subjects.

### 2.2 MfERG acquisition

**Fig. 2:**
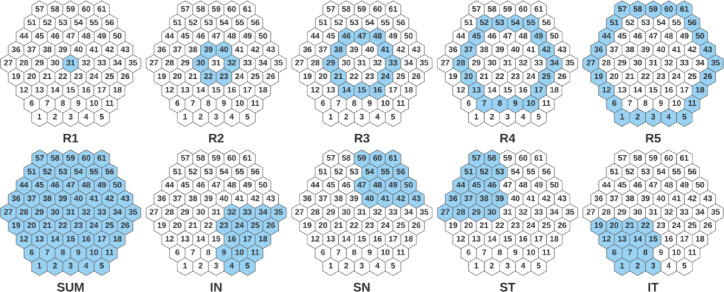
Definition of groups of mfERG responses. R1,…R5: Ring 1,…Ring 5; SUM: whole field; IN: Inferior Nasal; SN: Superior Nasal; ST: Superior Temporal; IT: Inferior Temporal.

The mfERGs were recorded using the RETI-port/scan 21 (Roland Consult, Berlin, Germany) visual electrophysiology system, according to the International Society for Clinical Electrophysiology of Vision (ISCEV) standard [31]. After full dilatation of the pupil and topical anaesthesia, a Dawson–Trick–Litzkow electrode was placed on the lower eyelid conjunctiva. The reference electrode was placed on a temple and the ground electrode on the nasion. A contact impedance of less than 10 KΩ was achieved. The fellow eye was occluded. The stimulus array consisted of 61 hexagonal sectors (S=1…61) (Fig. 2) displayed at a frame rate of 60 Hz. The luminance of each hexagon was independently alternated between black (< 2cd/m^2^ of luminance) and white (200 cd/m^2^ of luminance) according to a pseudorandom binary m-sequence [17]. To improve fixation stability, sessions were broken down into 47-s segments and 8 trials were recorded in total. An amplifier with a gain of 10^4^ and a bandwidth of 10–200 Hz was used. The signals were digitized at a sample rate of 1017 samples/s, with the number of samples from each signal being 84 (n = 1…84) (82.61 ms long). The first-order mfERG kernel was analysed.

### 2.3 MfERG grouping

According to the ISCEV standard [31], groups of mfERG responses from the sectors can be averaged to compare quadrants, hemiretinal areas or successive rings from centre to periphery. In our study, we considered the following 10 groups (Fig. 2): SUM (whole macular field), five concentric rings centred on the fovea for analysis from inner to outer: R1 (1 hexagon), R2 (6 hexagons, parafoveal ring), R3 (12 hexagons), R4 (18 hexagons) and R5 (24 hexagons); and four quadrants: superior temporal (ST), inferior temporal (IT), inferior nasal (IN) and superior nasal (SN). For each eye, the 10 described groupings were obtained by averaging the mfERG sectors that make up each grouping.

### 2.4 Feature extraction

**Fig. 3:**
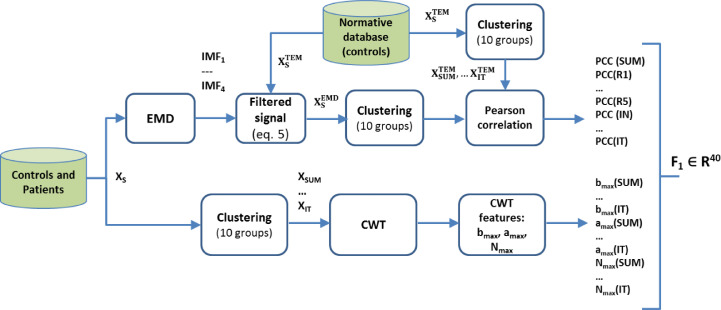
Feature extraction pipeline for F_1_ vector. EMD, empirical mode decomposition; IMF, intrinsic mode functions; CWT, continuous wavelet transform; PCC, Pearson correlation coefficient.

Two types of feature are used in mfERG signal classification. The first is the value of the correlation of the signals filtered by an adaptive filter based on EMD [29], while the second family of features is obtained from wavelet decomposition of the mfERG recordings (Fig. 3).

#### 2.4.1 EMD adaptive filter + correlation with a normative database

The method described in [29] comprises two stages (Fig. 3): a) adaptive filtering of the mfERG recordings using EMD and b) obtaining of the correlation coefficient between the filtered signal and a normative database. Briefly:

a) A normative database ($X^{\text{TEM}}$) was built for the original signals (RAW) and for each of the sectors (S = 1 … 61), averaging the traces of the 6 control subjects (Eq. (1)).
  $$X_{S}^{\text{TEM}}\left(n\right)=\frac{1}{6}\sum_{i=1}^{6}x\left(n\right);$$
  (1) S=1…61;n=1…84;
  When analysing the eye of a control subject, its signals were not included in the template. Thus, the template used to evaluate control subject j (j = 1 … 6) was as follows:
  $$X_{S}^{\text{TEM},\;j}\left(n\right)=\frac{1}{6-1}\sum_{i=1}^{6}x\left(n\right);$$
  (2) i≠j,S=1…61;n=1…84;
  Therefore, there is one template for analysis of the control subjects and another for analysis of MS patient recordings. The signals are grouped according to Fig. 2, obtaining a pattern for each of the 10 regions analysed: $X_{\text{SUM}}^{\text{TEM}},{\;X}_{R1}^{\text{TEM}},\;\ldots X_{\text{IT}}^{\text{TEM}}.$
b) Each signal (x(n)) corresponding to each of the sectors of the visual field is decomposed into a linear combination of L oscillating intrinsic mode functions (IMFs) plus a residue according to the original EMD method described in Huang et al. [32]:
  (3) $$x\left(n\right)=\sum_{k=1}^{L}\text{IM}F_{k}\left(n\right)+\text{residu}e_{L}\left(n\right)$$
  The first IMFs (k = 1, 2, …) represent the high-frequency components of x(n), while the higher IMFs (k = L, L−1, …) show the low-frequency information.
c) For each of the 61 sectors of the visual field, 4 approximations of the original signal are obtained, considering the contribution of the 4 IMFs into which it has been decomposed.
  $$x_{S}^{k}\left(n\right)=\sum_{k}^{4}\text{IM}F_{k}\left(n\right)+\text{residu}e_{L=4}\left(n\right);$$
  (4) S=1…61;k=1…4;n=1…84;
  The filtered signal ($X^{\text{EMD}}$) is the one among the 4 approximations obtained in Eq. (4) that has the highest Pearson correlation coefficient with the signal from the same sector of the normative database. Therefore, the filtered signal corresponding to sector S (S = 1 … 61) is Eq. (5):
  (5) $$X_{S}^{\text{EMD}}\left(n\right)=x_{S}^{k}\left(n\right)|\text{max}\;\text{Pearso}n_{\text{Corr}\left(X_{S}^{\text{TEM}}\left(n\right),x_{S}^{k}\left(n\right)\right)};k=1\ldots 4$$
d) The filtered signals are grouped as per Fig. 2 and, as the feature for characterization of the signals in each of the groups, the Pearson correlation coefficient (PCC) with that same group in the normative database is calculated. For example, the feature obtained for Ring 2 is as follows:
  (6) $$\text{PCC}\left(R2\right)=\;\text{Pearson}\_\text{Corr}\left(X_{R2}^{\text{TEMP}},{\;X}_{R2}^{\text{EMD}}\right);\;$$

#### 2.4.2 Wavelet signal analysis

The CWT converts a one-dimensional signal, x(t), into a two-dimensional function, T(a,b), in which it is possible to determine the frequency features at any given time. The continuous wavelet transform of x(t) is defined as:

(7) $$T\left(a,b\right)=\frac{1}{\sqrt{a}}\int_{-\infty }^{+\infty }x\left(t\right)\Psi^{*}\left(\frac{t-b}{a}\right)\text{dt}$$

where Ψ*(t) is the complex conjugate of wavelet function Ψ(t); *a* is the dilation parameter of the wavelet; and *b* is the translation parameter (a,b ∈ R; a ≠ 0). Wavelet transform T(a,b) is a two-dimensional function that shows the correlation (inner product) between the signal, x(t), and the wavelet function at different scales (a) and time instants (b). As the CWT can describe time and frequency components of a signal in detail, it is possible to obtain new mfERG signal descriptors that could be electrophysiological biomarkers of use in diagnosis of MS.

The CWT was applied to each of the 210 signals (21 eyes, 10 signals each) in the 0 ≤ b ≤ 82.61 ms time interval for scale 1 ≤ a ≤ 100. Repeated testing (Daubechies 7, Biorthogonal 3.1 and real Morlet [33]) showed that the real Daubechies 7 wavelet achieved the best discrimination between control subjects and patients.

### 2.5 CWT domain features

Different features can be obtained in the CWT domain (mean, max., min., entropy, etc.). For example, the scalogram can show the energy content of the signal in its different scales and time intervals. In other cases, the CWT coefficients of a certain scale are used directly as inputs of a neural network for their classification [34]. The scale of maximum correlation (a_max_) corresponds to the scale in which the maximum correlation value (max|T(a,b)|) appears between the signal, x(t), and the wavelet function. In [25], the coefficients of this scale (a_MAX_) are used as inputs of a radial-basis-function neural network for diagnosis of glaucoma, also using mfERG signals.

In the present study, the following variables obtained from the CWT of the mfERG recordings are considered:

a Time **b_MAX_** at which the absolute maximum value appears.
b Scale of maximum correlation (**a_MAX_**). Therefore: |T(a_MAX_,b_MAX_)| ≥ |T(a,b)|. This is the ‘a’ scale of the CWT in which the maximum |CWT| value is found.
c Number of local maxima (**N_MAX_**) in |T(a,b)| that exceed |T(a_MAX_, b_MAX_)|/3.

The three variables obtained in the CWT domain are calculated for each of the 10 visual field groups.

### 2.6 Feature ranking and selection

Feature selection methods can be divided into three main categories: filter methods, wrapper methods and embedded approach methods [35,36]. In the feature filter approach, ranking and dimensionality reduction are performed while taking into account certain intrinsic properties of each feature (e.g. variance, mutual information, discriminant capacity [37]) or even the knowledge of the medical expert who evaluates the suitability of certain features with respect to the neuroanatomy of the disease [38] independent of the classifier used. In the wrapper approach, the performance of the classifier is evaluated together with the input features. In the embedded approach, the classifier performs feature selection as part of the learning procedure. In [39], six types of feature selection method are identified: filter, wrapper, embedded, hybrid, ensemble and integrative, highlighting that the first three are the most widely used.

In our database, for each subject we initially have a feature vector **F**_1_ consisting of 40 features ($F_{1}\;\in \;R^{40}$; 4 variables x 10 visual field groups). A two-stage feature selection method was implemented (Fig. 1):

i) **F_1_** dimension reducibility using a feature filter approach. The initially relevant features are selected individually based on their capacity to discriminate between patients and control subjects, as evaluated by the area under the receiver operating characteristic curve (AUC). The output of this stage is a 4-variable feature vector **F_2_** ($F_{2}\;\in R^{4}$).
ii) The final architecture of CAD is determined using a wrapper approach, which takes into account the performance of the classifier. As the subset of features analysed in this stage is small $(F_{2}\;\in R^{4}$), it is possible to analyse all the cases and determine both the combination of final features and the SVM classifier kernel.

### 2.7 Automatic classifier

Since the purpose of this paper is to implement a classifier to aid diagnosis, after comparing various methods (multilayer perceptrons and radial basis function neural networks) we found that an SVM produces the best results. This classifier is characterized by being less sensitive to sample size than others and therefore potentially able to obtain useful results from a lower number of samples [40,41].

In a two-class classification problem, an SVM looks for the hyperplane that separates two different classes with maximum margin (support vectors) [42]. If the original data (**X**) are not linearly separable, a non-linear transformation may be performed to get a higher dimensional space (H) using a kernel function Φ(.):=X→H to improve the separability between the two classes in H. Kernel functions may be linear, polynomial (quadratic, cubic, etc.) or Gaussian.

Since the database is small, the predictive power of each SVM was evaluated using cross-validation with a leave-one-out procedure. Classification performance was assessed by using the Matthews Correlation Coefficient (MCC) [43]; MCC ranges are between +1 and -1; MCC = +1 represents perfect classification, MCC = -1 represents totally erroneous classification and MCC = 0 indicates a random prediction. In a binary classification problem, MCC is preferable over other parameters such as accuracy and F1 score [44]; it is also known to perform well with imbalanced data [45,46]. The MCC is defined as:

(8) $$\text{MCC}=\frac{\text{TP}\cdot \text{TN}-\text{FP}\cdot \text{FN}}{{\left(\left(\text{TP}+\text{FP}\right)\cdot \left(\text{TP}+\text{FN}\right)\cdot \left(\text{TN}+\text{FP}\right)\cdot \left(\text{TN}+\text{FN}\right)\right)}^{1\;/\;2}}$$

where TP (True Positive), TN (True Negative), FP (False Positive) and FN (False Negative) are the values obtained from the confusion matrix.

### 2.8 Statistical methods

Statistical analyses were performed with IBM SPSS Statistics 25 software (SPSS Inc. Chicago, Illinois, USA). The Shapiro–Wilk test was used for analysis of normality. Normally distributed variables are expressed as mean ± standard deviation; non-normally distributed variables are reported as median (interquartile range [IQR]). Comparison of groups was performed using Student's t-test (normal distributions) and the Mann–Whitney–Wilcoxon test (U) (not normal distributions). A p value ≤ 0.05 was considered statistically significant.

The area under the receiver operating characteristic curve (AUC) was employed to assess the discrimination capability for each of the features proposed in this study.

## 3 Results

MfERG recordings from 15 patients (age: 44.46 ± 8.24 years, male:female = 3:12, n = 15 eyes) with newly diagnosed MS (less than 6 months) and no history of optic neuritis, and those from 6 control subjects (age: 35.83 ± 10.65, male:female = 3:3, n = 6 eyes), were used. Normality of age in control subjects and MS patients was assessed using the Shapiro–Wilk test: p_CONTROL_= 0.108, p_MS_= 0.806. There was no significant difference between control subjects and MS patients in age: t(19) = -1.998, p = 0.060. In MS patients, overall disability measured by the Kurtzke Expanded Disability Status Scale (EDSS) [47] stood at 1.33 ± 0.54 and only 40% were in treatment (with varying MS therapies).

**Fig. 4:**
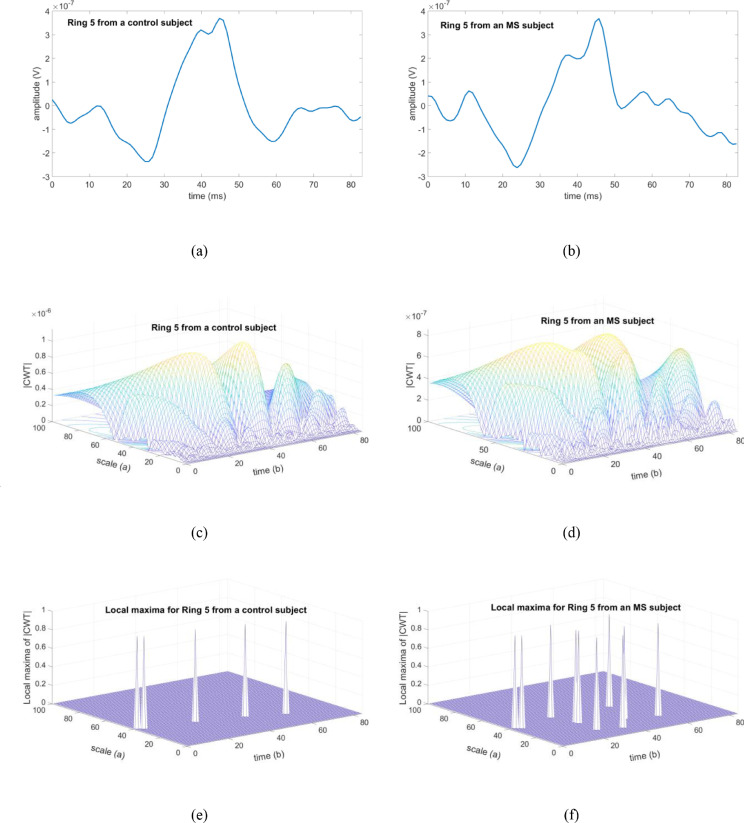
Examples of results for a control eye and an eye with MS, analysing Ring 5. (**a**) Recording from a control subject. (**b**) Recording from an MS patient. (**c**) |T(a,b)| for the control recording. (**d**) |T(a,b)| for the MS recording. (**e**) Local maxima of |T(a,b)| for the control recording. (**f**) Local maxima of |T(a,b)| for the MS recording. CWT: continuous wavelet transform.

As an example, Fig. 4 shows the signals recorded from a control subject (Fig. 4a) and an MS patient (Fig. 4b) corresponding to the Ring 5 grouping; the module of the CWT (Fig. 4c, d) and the results of the variable N_max_ (Fig. 4e, f) are also shown.

Table 1 shows the variables previously defined for the different mfERG recording groupings, for the eyes of the control subjects and for the MS patients (**F_1_** vector). In addition, the potential capacity to discriminate between control subjects and patients is indicated with the AUC value for all the variables and groupings.

**Table 1:** Values of variables defined for the different groupings of the mfERG recordings. C: controls; MS: multiple sclerosis; AUC: area under receiver operating characteristic curve; PCC: Pearson correlation coefficient; b_MAX_: time to the absolute maximum value; a_MAX_: scale of maximum correlation; N_MAX_: number of local maxima; SUM: whole field; Quad: quadrant; NI: nasal inferior; NS: nasal superior; TS: temporal superior; TI: temporal inferior. Values expressed as mean ± standard deviation (normally distributed) or median (interquartile range [IQR]) (non-normally distributed).

	**PCC**		**b_MAX_**		**a_MAX_**		**N_MAX_**		
	**C**n = 6	**MS**n = 15	**C**n = 6	**MS**n = 15	**C**n = 6	**MS**n = 15	**C**n = 6	**MS**n = 15	
**SUM**	0.98 ± 0.02	0.91 (0.89–0.95)	43.50 (37.75–45.00)	44.00 (43–47)	29.33 ± 3.82	30 (27–32)	4.5 (4–6.25)	7.40 ± 1.84	
	AUC = 0.91		AUC = 0.66		AUC = 0.57		AUC = 0.76		$\bar{\text{AU}C_{\text{SUM}}}=0.72$
**Ring 1**	0.95 ± 0.03	0.86 (0.71–0.94)	53.00 ± 1.26	52 (50–53)	38.0 (37.25–38.25)	39 (33–42)	3.00 ± 0.0	4 (3–5)	
	AUC = 0.83		AUC = 0.61		AUC = 0.56		AUC = 0.77		$\bar{\text{AU}C_{R1}}=0.72$
**Ring 2**	0.97 (0.92–0.98)	0.91 (0.71–0.94)	47.33 ± 1.36	43 (41–46)	31.33 ± 4.03	34.46 ± 5.35	7.33 ± 1.75	7.60 ± 2.92	
	AUC = 0.87		AUC = 0.83		AUC = 0.63		AUC = 0.53		$\bar{\text{AU}C_{R2}}=0.68$
**Ring 3**	0.96 ± 0.03	0.90 (0.75–0.94)	44.33 ± 1.03	43 (42–42)	31.16 ± 6.11	29.66 ± 3.06	5.83 ± 2.40	6.0 (5–7)	
	AUC = 0.89		AUC = 0.73		AUC = 0.54		AUC = 0.64		$\bar{\text{AU}C_{R3}}=0.70$
**Ring 4**	0.95 ± 0.02	0.86 (0.68–0.90)	43.00 (37.75–44.25)	44 (42–46)	28.33 ± 2.42	33.53 ± 7.94	5.00 ± 1.26	8.20 ± 2.65	
	AUC = 0.96		AUC = 0.67		AUC = 0.68		AUC = 0.86		$\bar{\text{AU}C_{R4}}=0.79$
**Ring 5**	0.95 (0.93–0.97)	0.82 ± 0.11	44.50 ± 2.42	44 (41–46)	29.50 ± 5.54	30 (26–44)	5.50 ± 1.37	9.20 ± 2.80	
	AUC = 0.92		AUC = 0.58		AUC = 0.62		AUC = 0.89		$\bar{\text{AU}C_{R5}}=0.75$
**Quad IN**	0.97 ± 0.02	0.91 (0.82–0.93)	44.33 ± 2.58	43 (31–45)	32.50 ± 7.66	30 (26–38)	6.66 ± 1.86	8 (6–11)	
	AUC = 0.94		AUC = 0.67		AUC = 0.51		AUC = 0.67		$\bar{\text{AU}C_{\text{IN}}}=0.70$
**Quad SN**	0.96 ± 0.01	0.85 (0.63–0.93)	44.50 (38.25–45.25)	44 (27–46)	27.50 (26.00–33.25)	35.33 ± 7.51	6.33 ± 1.96	8.40 ± 3.52	
	AUC = 0.92		AUC = 0.52		AUC = 0.75		AUC = 0.67		$\bar{\text{AU}C_{\text{SN}}}=0.71$
**Quad ST**	0.96 ± 0.02	0.83 ± 0.13	44.00 ± 1.09	44 (42–47)	27.16 ± 2.13	30.40 ± 5.77	7.16 ± 2.22	6 (4–12)	
	AUC = 0.91		AUC Z 0.50		AUC = 0.71		AUC = 0.50		$\bar{\text{AU}C_{\text{ST}}}=0.65$
**Quad IT**	0.93 ± 0.04	0.83 (0.53–0.91)	43.5 (36.25–45.25)	43 (42–46)	28.33 ± 3.88	31 (26–40)	7.50 ± 3.20	10 (8–12)	
	AUC = 0.88		AUC = 0.56		AUC = 0.64		AUC = 0.72		$\bar{\text{AU}C_{\text{IT}}}=0.70$
	$\bar{\text{AU}C_{\text{PCC}}}=0.90$		$\bar{\text{AU}C_{b_{MAX}}}=0.61$		$\bar{\text{AU}C_{a_{MAX}}}=0.62$		$\bar{\text{AU}C_{N_{MAX}}}=0.70$		

The filter feature selection method implemented is based on first determining the groupings that, based on the mean, have the highest discriminant value, these being R4 ($\bar{\text{AU}C_{R4}=0.79}$) and R5 ($\bar{\text{AU}C_{R5}=0.75}$) (Table 1). The parameters that, based on the mean, have the highest AUC value are identified, these being PCC ($\bar{\text{AU}C_{\text{PCC}}}=0.90$) and N_MAX_ ($\bar{\text{AU}C_{N_{\text{MAX}}}}=0.70$) as the mean AUC value of the other two variables is much lower in relative terms (Table 1). Therefore, the output of the initial feature-filtering process is **F_2_** = (PCC(R4), PCC(R5), N_MAX_(R4), N_MAX_(R5), resulting in a dimensionality reduction from 40 to 4 features.

**Fig. 5:**
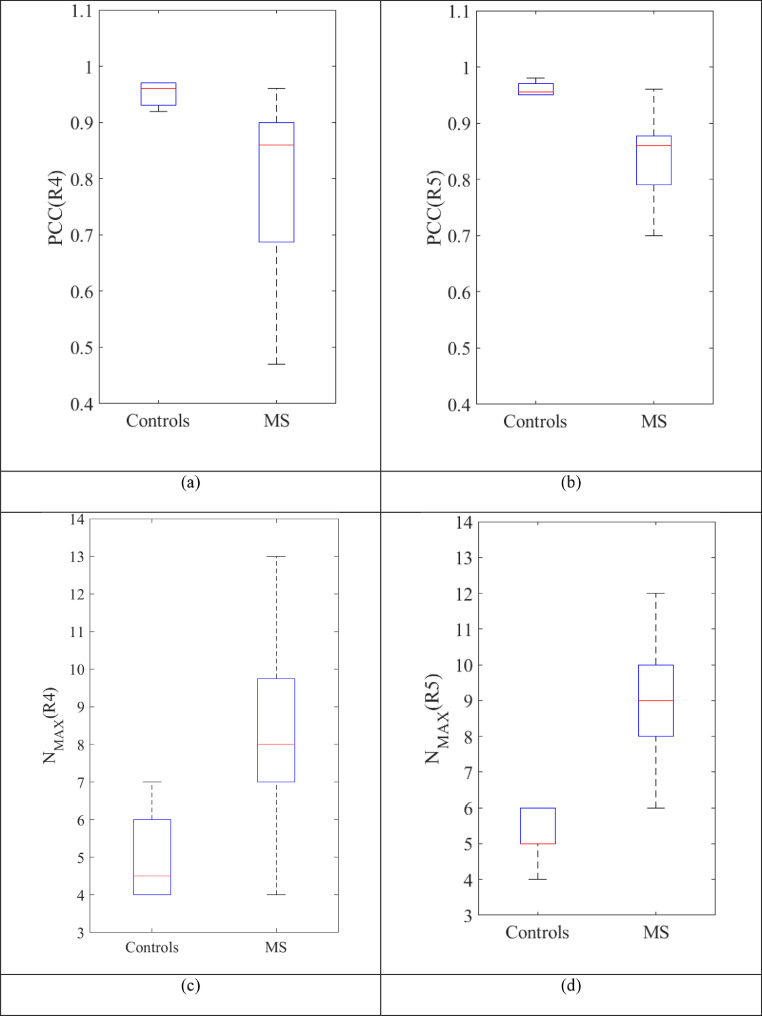
Box plot of the variables that make up the feature vector F_2_. (a) PCC (R4). (b) PCC (R5). (c) N_MAX_ (R4). (**d**) N_MAX_ (R5). MS: multiple sclerosis.

The PCC(R4) and PCC(R5) variables are statistically significantly higher in the control group than the MS group: PCC(R4) (Mann–Whitney U = 5.0, p = 0.02), PCC(R5): (Mann–Whitney U = 7.5, p = 0.03). Nevertheless, N_MAX_(R4) and N_MAX_(R5) are higher in the MS group: N_MAX_(R4) (t-test, difference in means is 3.2, p = 0.011, 95% CI: 0.81 to 5.59, equal variances assumed) and N_MAX_ (R5) (t-test, difference in means is 3.70, p = 0.007, 95% CI: 1.16 to 6.24, equal variances assumed) (Fig. 5).

In the second-stage feature selection method, a wrapper approach evaluates the overall behaviour of the CAD, taking into account all possible combinations of the 4 pre-selected variables and using the MCC value, obtained from the corresponding confusion matrix, as a measure of the classifier's performance. Training and evaluation are performed using cross-validation with a leave-one-out procedure, and different types of SVM kernels: linear ($\Phi \left(x_{i},x_{j}\right)={x_{i}}^{T}x_{j}$), cubic $\left(\Phi \left(x_{i},x_{j}\right)={\left(x_{i}^{T}x_{j}+1\right)}^{3}\right)$, Gaussian $\left(\Phi \left(x_{i},x_{j}\right)=\text{exp}\left(-\frac{{∥x_{i}-x_{j}∥}^{2}}{2\sigma^{2}}\right)\right)$ have been tested to map the input data vector into higher dimensional spaces; the mapped feature vectors can be linearly separable or have improved separability.

Table 2 shows the results of the different versions of the CAD implemented. For each combination of features considered (in the table, ‘1’ indicates inclusion and ‘0’ indicates exclusion), the results corresponding to the best MCC are presented, indicating the SVM kernel. For each of the input combinations, the hyperparameters that obtain the best classification are kernel function, kernel scale (in Gaussian kernels) and the box constraint parameter or soft-margin penalty (C).

**Table 2:** Evaluation of the different classifier configurations. C: Box Constraint parameter or soft-margin penalty. σ= width of the Gaussian kernel. TP, True Positive, TN, True Negative, FP, False Positive; FN, False Negative, MCC, Matthews Correlation Coefficient.

FEATURES (F_2_)				SVM hyperparameters	Confusion Matrix				MCC
PCC(R4)	PCC(R5)	N_MAX_ (R4)	N_MAX_ (R5)		TN	FP	FN	TP	
0	0	0	1	Kernel: Linear C=2	5	1	1	14	0.77
0	0	1	0	Kernel: Linear C=2	5	1	3	12	0.58
0	0	1	1	Kernel: Gaussian C = 2 σ =2.4	6	0	2	13	0.81
0	1	0	0	Kernel:Gaussian C=4 σ =0.25	5	1	2	13	0.67
0	1	0	1	Kernel: Gaussian C=2 σ =1.4	5	1	1	14	0.76
0	1	1	0	Kernel:Gaussian C=2 σ =1.4	6	0	1	14	0.89
0	1	1	1	Kernel:Gaussian C=2 σ =1.7	6	0	2	13	0.81
1	0	0	0	Kernel: Linear C=4	6	0	2	13	0.81
1	0	0	1	Kernel: Cubic C=2	5	1	2	13	0.67
1	0	1	0	Kernel: Linear C=2	6	0	1	14	0.89
1	0	1	1	Kernel:Gaussian C=3 σ =1.7	6	0	2	13	0.81
1	1	0	0	Kernel: Cubic C=2	6	0	3	12	0.73
1	1	0	1	Kernel: Gaussian C=1 σ =0.5	5	1	0	15	0.88
1	1	1	0	Kernel: Gaussian C=2 σ =1.7	6	0	2	13	0.81
1	1	1	1	Kernel: Cubic C=2	6	0	2	13	0.81

As can be seen, when only one variable (0001, 0010, 0100, 1000) is considered, the best MCC value is 0.77; when considering a feature vector comprising 4 variables (1111), the MCC value is 0.81. The best results in the classification are obtained by considering two inputs: PCC(R5) and N_MAX_(R4) (SVM kernel: Gaussian) or the PCC(R4) and N_MAX_(R4) pair (SVM kernel: linear), in both cases with the same confusion matrix and MCC = 0.89. The rate of correct predictions (accuracy) is 0.95, the ability to detect controls (specificity) is 1.0 and the sensitivity of the best CAD configuration is 0.93, indicating reliable detection of MS.

## 4 Discussion

Obtaining new MS biomarkers will make it possible to achieve early and more accurate diagnosis, predict disease progression and facilitate patient stratification in clinical and therapeutic research settings. Moreover, if these new biomarkers are based on low-cost, non-invasive tests, patient handling will be improved cost-effectively and performance of other invasive and more discomforting tests will be reduced. The purpose of this paper has been to research application of mfERG recordings to contribute to early diagnosis of MS in patients with no history of optic neuritis.

Not many papers have used analysis of mfERG recordings to study the outer retinal function in patients with MS and the findings of them are inconclusive. To the best of our knowledge, all cases are based on traditional analysis of wave amplitudes and latencies [[18], [19], [20],^22^]. Several studies propose using the latency of the P1 wave to evaluate MS, among them [21], which uses the latency of the P1 wave in the parafovea as a marker of MS progression. More recently, [23] observed that the latency of the P1 wave increased in patients with MS (independently of ON).

This paper researches, for the first time, a CAD system for MS diagnosis based on the analysis of mfERG recordings and the fusion of two types of feature: correlation of EMD-filtered signals with a normative database and features obtained from the continuous wavelet transform domain. Using a fusion approach, it is possible to obtain a classifier with high precision values.

We have shown significant electroretinographic changes of the outer retina neurons in patients with early MS and no history of ON, applying an innovative set of signal-analysis tools to the mfERG responses. Our results suggest that the effects of MS on the outer retina may be a more common dysfunction than previously anticipated and that it could be used as an electrophysiological biomarker for MS activity. Our findings show high AUC values and have the particular characteristic of being obtained in a population of recently diagnosed MS patients in which the disease is not severe and who do not have a history of optic neuritis, i.e. in patients in which affectation of the visual pathway by MS is expected to be minimal. The impairment detected in the outer retina in the initial stages of the disease could be used in early detection, perhaps even as a test in rapid MS diagnosis programmes, given that it would expedite diagnosis and would be less dependent on identifying changes in serial MRI recordings (as occurs with current MS diagnostic criteria). This test has several advantages over MRI: it is innocuous, low-cost and relatively fast.

With our analysis method, the greatest difference between control subjects and patients presents in Rings 4 and 5. The mfERG topographically evaluates the macular and paramacular retina under photopic conditions, and therefore the cone activity. Rings 4 and 5 are located in the mid-periphery, where cone-photoreceptor density diminishes considerably. In this zone, a small loss of cone functionality is therefore easier to detect than in other zones with greater cone density (such as, for example, the fovea). This may be the reason why Ring 5 presents greater affectation in the incipient stages of MS in which the loss of functionality is still very slight. Our findings therefore indicate that the photoreceptors present a form of susceptibility (individual, antigenic or inflammatory) that leads to their dysfunctionality in the early stages of MS [48]. Another possibility is that their susceptibility is caused by the toxic effect of the treatment prescribed. However, in our patient sample, only 40% received treatment and those that did receive it received different drugs. We therefore do not consider this to be a factor influencing cone functionality.

The topographic zone of the retina (mid-periphery and paramacular), corresponding to 15–20° (Ring 4 and Ring 5 of the mfERG), is principally populated by parasol and midget retinal ganglion cells (RGCs) responsible, among other functions, for processing low- and high-frequency vision and red-green colour, respectively [49], to send the information to the brain. Alteration of these RGCs is supported by papers that observe affectation of sensitivity to low- and high-frequency contrast, as well as slight protanomaly (red colour deficiency) in the early stages of MS as a consequence of axonal damage [50,51]. These RGCs receive input from the paramacular cones, which are the receptor cells initially responsible for processing the red colour information. Thus, possible subclinical functional impairment of the paramacular/peripheral cones of the rearmost visual pathway (as detected in our study) could be partly responsible for the colour alterations, even in the early stages of MS without optic neuritis. These visual disturbances could be easily explored using the mfERG test.

Our results suggest that the effects of MS on the outer retina neurons [23] and bipolar connections [52,53] may be a more common anomaly than once believed, as other authors described. A possible role for glutamate in abnormal outer retinal function in MS patients has even been suggested [23]*.* This glutamate-induced cell damage contributes to neural dysfunction and then to the electroretinographic changes of the photoreceptor and bipolar cell synapses detected by the mfERG*.*

Our outer retinal dysfunction results do not appear to be a consequence of demyelination and inflammation, as our subjects were MS patients without ON. Based on the current evidence, it is very likely that in our MS subjects there is a long delay in development of changes that are structurally detectable with OCT compared with the earlier and more subtle functional changes in the outer retina detected with mfERG in our study, as [23] also reported.

A previous study has demonstrated the usefulness of CAD techniques in detecting neurological abnormalities and improving the consistency of diagnosis and treatment using electroencephalograms (14 channels) in MS patients with less than 3 years’ average disease duration [54]. Our study has the advantage that it uses multifocal ERG, which is a non-invasive test that is much more comfortable for patients. Development of CAD to aid MS diagnosis has advanced recently, but these systems should be minimally invasive, and reserved for newly diagnosed patients.

Another study conducted by our research group confirms that it is possible to classify control subjects and MS patients without previous optic neuritis by applying machine-learning techniques to analysis of structural neurodegeneration in the retina (based on optical coherence tomography measurements) [55]. Our findings suggest that there is evidence of subclinical changes in both the structure and functionality of the visual pathway in patients in early stages of MS.

Further cross-sectional studies with a larger cohort of MS patients will be required to confirm whether the degree of outer retinal dysfunction as detected by our analysis is related to MS activity and progression. Analysis using pattern ERG and multifocal PEV may also help to locate initial impairment to the visual pathway topographically in MS patients [56].

The most common method for diagnosing MS is contrast MRI, which is an invasive test that incurs a high cost for the health service. Furthermore, it is usually necessary to perform a series of MRI scans to reach a definitive diagnosis. If the findings of our research are confirmed in a more extensive study (greater number of subjects, inter-centre, etc.), our method could constitute an innocuous complementary option that would help reduce healthcare expenditure. It would also be useful to research the utility of other clinical or ophthalmological markers (such as OCT thicknesses) that, when used jointly with mfERG, could increase the reliability of the diagnosis and offer a cost-effective alternative [57]. In addition, it would be useful to analyse the multifocal ERGs of subjects with other neurological conditions in order to demonstrate that the proposed tool is able to discriminate not only MS patients from healthy control subjects, but also from subjects with other central nervous system diseases.

The main strength of this study lies in the fact that the subjects evaluated presented very slight MS, which makes it difficult to differentiate these patients from healthy subjects. Despite this similarity, the classifiers described in this paper obtained a high AUC and a very low rate of false negatives. This demonstrates the utility of the method, even in challenging cases in patients in which the symptoms of MS are only starting to present or which have yet to receive definitive diagnosis.

EMD is one of the mathematical tools used to analyse mfERG recordings. When working with intermittent signals containing noise, mode mixing (the existence of more than one timescale in a single IMF or the same timescale occurring in more than one IMF) can be a problem. This study has used the original version of the algorithm [32] with the piecewise cubic Hermite interpolating polynomial approach that reduces overshooting. The algorithm has performed well, partly due to the fact that the analogue signals are filtered with a bandwidth of 10–200 Hz and because the EMD algorithm is applied to averaged signals (whole visual field, rings, quadrants). Nevertheless, it is still advisable to evaluate the performance of the CAD with subsequent enhancements, such as ensemble empirical mode decomposition (EEMD) [58], complete ensemble empirical mode decomposition with adaptive noise (CEEMDAN) [59] or improved CEEMDAN [60], among other options.

The main limitations of our study are the small size of our database and the lack of balance within it (15 patients, 6 controls). To avoid inter-eye correlation in the statistical study, only one eye from each subject was selected. Another limitation is that patients and controls have not been followed up. To avoid bias deriving from the differences between databases, the database should be extended to include subjects from other centres and signals recorded using other commercially available equipment. Although the sample is small, the population is well characterized and the inclusion criteria were exhaustive so as to avoid including possible subclinical glaucoma or other silent pathologies that could influence the findings.

## 5 Conclusions

This study shows an alteration of the outer retina in patients with incipient MS and without optic neuritis detected by mfERG recordings. The most important finding of this paper is the ability to differentiate between control subjects and MS patients in the early stages of the disease, fusioning two types of feature and using an SVM as a classifier.

## Author statement

A. López-Dorado^,^ L. Boquete, E. Garcia-Martin: Conceptualization; J. Pérez, J. M. Miguel-Jiménez, M. Ortiz, L. de Santiago, E. López-Guillén, R. Blanco, C. Cavalliere: Data curation; J. M. Miguel-Jiménez, M. Ortiz, L. de Santiago, E. López-Guillén, R. Blanco, C. Cavalliere: Formal analysis; L. Boquete, E. Garcia-Martin: Funding acquisition; A. López-Dorado^,^J. Pérez, J. M. Miguel-Jiménez, M. Ortiz, L. de Santiago, E. López-Guillén, R. Blanco, C. Cavalliere, E. Mª Sánchez Morla: Investigation; A. López-Dorado, J. Pérez, M. J. Rodrigo: Methodology; L. Boquete, E. Garcia-Martin: Project administration; L. Boquete, E. Garcia-Martin: Resources; A. López-Dorado, J. M. Miguel-Jiménez, M. Ortiz, L. de Santiago, E. López-Guillén, R. Blanco, C. Cavalliere: Software; L. Boquete, E. Garcia-Martin: Supervision; A. López-Dorado, M. J. Rodrigo, L. Boquete, E. Garcia-Martin: Validation and Visualization; M. J. Rodrigo, L. Boquete, E. Garcia-Martin: Roles/Writing - original draft; A. López-Dorado, M. J. Rodrigo, L. Boquete, E. Garcia-Martin: Writing - review & editing.

## Funding

Acknowledgements: This research was supported by the Secretariat of State for Research, Development and Innovation [grant number DPI2017-88438-R (AEI/FEDER, EU), awarded to LB], the Carlos III Health Institute [grant number PI17/01726 for “Neuro-ophthalmological evaluation as biomarker of diagnosis, monitoring and prognosis in multiple sclerosis”, awarded to EGM and RETICS Oftared, RD16/0008/020, awarded to LB and RD16/0008/029, awarded to EGM

## Declaration of Competing Interest

The authors declare that they have no known competing financial interests or personal relationships that could have appeared to influence the work reported in this paper.
